# Incidence of Mumps Deafness in Japan, 2005–2017: Analysis of Japanese Insurance Claims Database

**DOI:** 10.2188/jea.JE20200233

**Published:** 2022-01-05

**Authors:** Akira Takagi, Satoko Ohfuji, Takashi Nakano, Hideaki Kumihashi, Munehide Kano, Toshihiro Tanaka

**Affiliations:** 1 Department of Otorhinolaryngology, Head and Neck Surgery, Shizuoka General Hospital, Shizuoka, Japan; 2 Department of Public Health, Osaka City University Graduate School of Medicine and Research Center for Infectious Disease Sciences, Osaka City University Graduate School of Medicine, Osaka, Japan; 3 Department of Pediatrics, Kawasaki Medical School, Okayama, Japan; 4 Global Vaccine Business Unit, Takeda Pharmaceutical Company Limited, Tokyo, Japan; 5 Department of Pediatrics, Shizuoka Kosei Hospital, Shizuoka, Japan

**Keywords:** mumps deafness, congenital deafness, unilateral neurosensory deafness, mumps vaccine

## Abstract

**Background:**

Mumps deafness causes serious problems, and incidence data are needed to identify its disease burden. However, such data are limited, and the reported incidence is highly variable. Nationwide studies in Japan with a large age range are lacking.

**Methods:**

This was a retrospective observational investigation of the 2005–2017 mumps burden using employment-based health insurance claims data. Data were analyzed for 5,190,326 people aged 0–64 years to estimate the incidence of mumps deafness.

**Results:**

Of 68,112 patients with mumps (36,423 males; 31,689 females), 102 (48 males; 54 females) developed mumps deafness—an incidence of 15.0 per 10,000 patients (1 in 668 patients). Fifty-four (52.9%) patients had mumps deafness in childhood (0–15 years), and 48 (47.1%) had mumps deafness in adolescence and adulthood (16–64 years); most cases occurred in childhood, the peak period for mumps onset. The incidence of mumps deafness per 10,000 patients was 73.6 in adolescence and adulthood, 8.4 times higher than the incidence of 8.8 in childhood (*P* < 0.001). In childhood, the incidence of mumps deafness was 7.2 times higher among 6–15-year-olds (13.8; 95% CI, 10.2–18.2) than among 0–5-year-olds (1.9; 95% CI, 0.6–4.5), and this difference was statistically significant (*P* < 0.001). No sex difference was observed.

**Conclusions:**

The incidence of mumps deafness per 10,000 patients aged 0–64 years was 15.0 (1 in 668 patients). A secondary risk of deafness following mumps virus infection was identified not only for children, but also for adolescents and adults.

## INTRODUCTION

Mumps is caused by the mumps virus. It typically starts with symptoms such as fever, headache, and muscle pain, followed by swelling of the parotid and/or submandibular glands. For most patients, mumps symptoms are quite mild, but some experience serious outcomes (such as meningitis, encephalitis, or deafness) that can result in long-term problems. To prevent mumps epidemics, several countries conduct routine mumps immunization to achieve a high vaccination rate. However, mumps epidemics sometimes occur in Japan because of the low mumps immunization rate.

Routine immunization with the measles, mumps, and rubella (MMR) vaccine was implemented in 1989 in Japan. In 1993, MMR vaccination was discontinued owing to the high rate of aseptic meningitis caused by certain mumps vaccine strains.^1^ Monovalent mumps vaccination has been resumed on a voluntary basis, but the mumps immunization rate remains consistently low, at approximately 30%, resulting in nationwide mumps epidemics at intervals of 4–5 years.^2^

Mumps deafness is a serious, lifelong mumps complication that occurs from 4 days before to 18 days after the swelling of the parotid and/or submandibular glands.^3^ This condition is usually unilateral and incurable.^4^^,^^5^ There is no consensus on the frequency of mumps deafness in Japan; estimates vary greatly, from 1 in 18,000 patients^6^ to 1 in 1,057 patients^4^ or 1 in 294 patients^7^ with mumps. Nishioka et al reported an incidence of 1 in 18,000 using a questionnaire survey after the 1987 mumps epidemic in Ehime Prefecture.^6^ Hashimoto et al examined 7,400 patients with mumps and their hearing test records from January 2004 to December 2006^4^ and found an incidence of 1 in 1,057. Aoyagi et al reported an incidence of 1 in 294 using a retrospective survey conducted from October 1993 to September 1994 in Wakayama Prefecture.^7^ These three studies were conducted in limited areas from 1987 to 2006 and targeted individuals younger than 20 years. Taya et al reported that mumps deafness is found in both pediatric and adult mumps patients.^8^ However, no nationwide surveys of the incidence of mumps deafness in Japan have included adults. Everberg conducted a meta-analysis of global data on mumps deafness and reported that the incidence of suspected mumps deafness was about 1 in 20,000 in 1957.^9^

The Oto-Rhino-Laryngological Society of Japan conducted a nationwide survey of mumps deafness in 2015–2016 and reported that 348 patients had a definitive diagnosis of mumps deafness, which was bilateral in 16 patients.^10^ However, this survey did not address the incidence of mumps deafness in mumps patients because the proportion of patients with mumps infection in the target population is unknown. To obtain an up-to-date estimate of the incidence of mumps deafness in mumps patients, we examined data from a Japanese health insurance claims database.

## METHODS

### Study design and data source

This was a retrospective, observational database study. The data source was the Japanese Claims Database, which is an employment-based health insurance claims database that includes data for about 200 major Japanese enterprises. The database was developed by JMDC Inc. (Tokyo, Japan). The JMDC database standardizes the diagnosis names using the standard disease names provided by the Medical Information System Development Center and the codes for the electronic claims processing system. These standardized diagnosis names are then linked with the ICD-10 codes (2013 version). Medical practices including laboratory examinations are also standardized, and the database provides standardized medical practice names, codes, and medical fee point chart classification codes from the Ministry of Health, Labour and Welfare. The cumulative dataset comprises approximately 5.5 million subjects (as of June 2018) and accounts for about 4% of the total population of Japan, with records beginning in 2005. However, data for individuals aged >65 years are limited because the database is based on employment-based health insurance claims, and the average retirement age in Japan is 60–65 years. Considering the nature of this database (employment-based health insurance claims data), the study population may not be representative of the population of Japan in terms of the average income. Data can be tracked for subjects who have employment insurance targeted by the database even if they transfer between hospitals or use multiple facilities. In this study, data from January 2005 to December 2017 were analyzed. Because the study used pre-existing anonymized data, it was not necessary to obtain informed patient consent.

Two individuals from JMDC Inc. independently performed the data extraction from the database, program construction, and analyses. These individuals reached consensus on the data extraction process and study protocol, thus ensuring the quality of the data.

### Inclusion and exclusion criteria

Records for subjects registered in the Japanese Claims Database from January 2005 to December 2017 were screened for insured period and age. Subjects aged ≤64 years with ≥3 months of insurance coverage were included in this study. Subjects with a history of recurrent parotitis were excluded from the analysis.

To ensure observation over a full year, data provided from health insurance associations that maintained a contract with JMDC for consecutive data submission from January to December in a given year were included in the analyses; data from health insurance associations with contracts that covered less than 12 months per year were excluded. As for individuals, data on people aged ≤64 years who were confirmed as enrolled in a health insurance association for at least 3 months—regardless of at which point during the year they enrolled or withdrew from the relevant health insurance association—were included in the analysis.

### Definition of mumps

Mumps cases were diagnosed using the B26 code from the *International Statistical Classification of Diseases and Related Health Problems, 10^th^ Revision* (ICD-10). Subjects with mumps who met all the following criteria were excluded as suspected cases of recurrent parotitis: 1) a diagnosis of mumps during the observation period; 2) no diagnosis of mumps complications during the observation period; 3) diagnosis of parotitis within 6 months prior to the mumps diagnosis during the observation period; and 4) diagnosis of mumps or parotitis without complications within 2 to 6 months following the mumps diagnosis during the observation period. The mumps onset date was defined as the date of the first visit recorded, that is, the earliest medical treatment start date described in the diagnostic record during the observation period.

### Definition of mumps deafness

**Figure 1.**
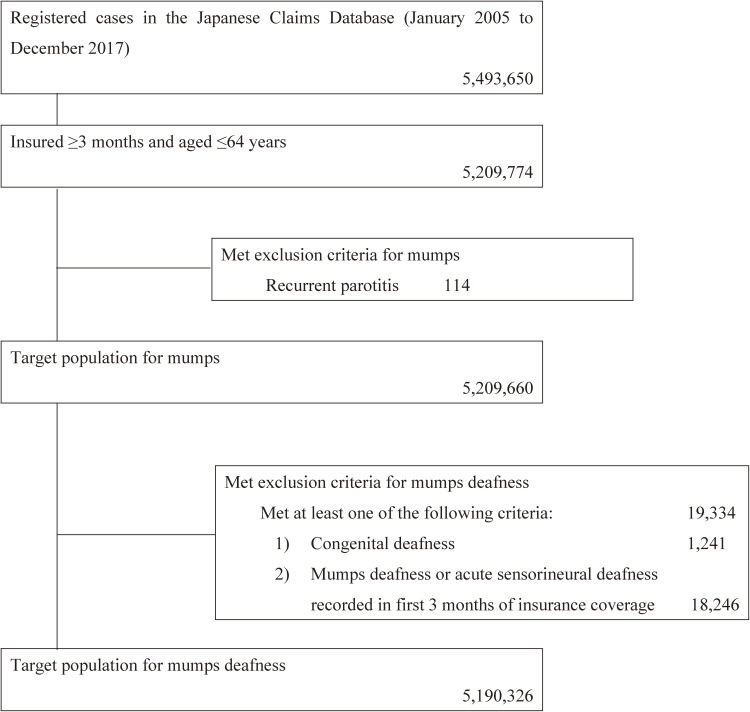
Target populations for mumps and mumps deafness

Relevant conditions and tests used to diagnose mumps deafness were identified using ICD-10 codes and ICD-10-based Standard Disease Code (eTable 1) and reimbursed laboratory tests (eTable 2). Mumps deafness (Classified ID: 20053371) (Figure 1) had to be diagnosed after mumps (ICD-10: B26) onset, as described in the next paragraph. To be diagnosed as having mumps deafness, cases could not have 1) a documented diagnosis of congenital deafness (20067696, 20067729, 20067843) within the observation time window or 2) a documented diagnosis of mumps deafness or acute sensorineural deafness (20098025, 20098024, 20077692, 20054502, 20079074, 20076891, 20066048, 20066139, 20056854, 20072135, 20061652, 20075882) within 3 months after the start of follow-up.

Mumps deafness (eFigure 1) was defined as deafness meeting any of the following criteria without a documented diagnosis of Meniere’s syndrome (20053401), Meniere’s disease (20087640, 20072146, 20072185, 20068157), ear herpes (20053049, 20063249), stenosis of Eustachian tube (20063320, 20063325, 20063331), delayed endolymphatic hydrops (20101275), Otitis media (ICD-10 second classification code: A18, A38, B05, H65, H66, H71, H74, H83, J11, T70), Congenital middle ear malformation (Classified ID: 20070494), otitis media with effusion (20058665, 20076669, 20078364), acoustic neuroma (ICD-10 third classification code: C07, C30.1, C49.0, C72.4) or cochlear Meniere’s disease (Classified ID: 20098854) in the same month: 1) a documented diagnosis of mumps deafness, with viral antibody titer determination according to immunoglobulin class (mumps virus) (Medical service fee code: 160157310) and/or standard pure tone audiometry (Medical service fee code: 160078010) performed in the same month; 2) onset of acute sensorineural deafness within 1 month after mumps onset, with viral antibody titer (mumps virus) determination according to immunoglobulin class (mumps virus) and/or standard pure tone audiometry performed in the same month; or 3) onset of acute sensorineural deafness and mumps antibody test (Medical service fee code: 160042410) to confirm mumps infection, and viral antibody titer determination according to immunoglobulin class (mumps virus) and/or standard pure tone audiometry performed in the same month as the diagnosis.

### Statistical analysis

The incidence of mumps deafness (per 10,000 cases) was defined as the number of people with mumps deafness/the number of patients with mumps × 10,000. The approximate confidence interval (CI) based on the Poisson distribution was calculated for the incidence over the study years (2005–2017). SAS, Version 9.4 (SAS Institute Japan Ltd., Tokyo, Japan) was used for the statistical analysis. A significance level of 1% was used in chi-squared tests by age group and by sex.

### Ethical approval and informed consent

Because the construction of the medical information database used in this study makes it impossible to identify individual patients, it was not possible to provide a direct explanation of the study to patients whose data were used; therefore, informed consent was not obtained from the study subjects. The *Ethical Guidelines for Medical and Health Research Involving Human Subjects*^11^ do not apply to research using only already established anonymized or de-identified information, as specified in Part 3, “Scope of Application,” in Chapter 1. Because the present study, which used only pre-existing anonymized information, was not subject to these guidelines and was deemed to pose no risk or disadvantage to the subjects, the study was not reviewed by an ethics board.

## RESULTS

The number of insured people in the database increased from 277,585 in 2005 to 3,827,283 in 2017. We analyzed data collected on 5,209,660 subjects (excluding 114 subjects with recurrent parotitis) over a 13-year period from 2005 to 2017 (Figure 1). For the final analysis of mumps deafness, we also excluded 19,334 cases with 1) congenital deafness (1,241 cases) or 2) mumps deafness or acute sensorineural deafness onset recorded in the first 3 months of the insurance period (18,246 cases). Data from a final total of 5,190,326 subjects were analyzed (Figure 1). Of these 5,190,326 subjects, 68,112 were identified as mumps cases.

As shown in Table 1, the total number of subjects identified as mumps cases in 2005–2017 was 68,112 (36,423 males [53.5%] and 31,689 females [46.5%]). Of these cases, 102 subjects (48 males and 54 females) developed mumps deafness. The incidence of mumps deafness per 10,000 mumps patients was 15.0 (1 in 668 patients). The peak onset age of mumps deafness was 6–15 years (49 patients, 48.0%), followed by 26–35 years (21 patients, 20.6%). Fifty-four cases had mumps deafness in childhood (age 0–15 years), accounting for 52.9% of all mumps deafness cases, and 48 (47.1%) had mumps deafness in adolescence and adulthood (age 16–64 years). The incidence of mumps deafness per 10,000 mumps cases was 8.4 times higher among 16–64-year-olds (73.6; 95% CI, 52.9–94.4) than among 0–15-year-olds (8.8; 95% CI, 6.4–11.1), and this difference was statistically significant (*P* < 0.001). In childhood, the incidence of mumps deafness was 7.2 times higher among 6–15-year-olds (13.8; 95% CI, 10.2–18.2) than among 0–5-year-olds (1.9; 95% CI, 0.6–4.5); this difference was also statistically significant (*P* < 0.001). The incidence of mumps deafness per 10,000 patients was 13.2 for males and 17.0 for females, and the difference between the two groups was not significant (*P* = 0.194).

**Table 1.** Incidence rates of mumps deafness in male and female mumps patients of a health insurance cohort in Japan, 2005–2017 (*N* = 5,190,326)

			Mumps deafness			

Sex	Age group, years	Mumps patients, *N*	Onset patients, *n*	Incidence per 10,000 mumps patients [95% confidence interval]		Incidence *N*/*n*
All	All ages	68,112	102	15.0	[12.2–18.2]	668
	0–5	25,999	5	1.9	[0.6–4.5]	5,200
	6–15	35,595	49	13.8	[10.2–18.2]	726
	(0–20)	(62,551)	(57)	(9.1)	(6.9–11.8)	(1,097)
	16–25	1,518	10	65.9	[31.6–120.8]	152
	26–35	2,405	21	87.3	[54.1–133.2]	115
	36–45	1,893	9	47.5	[21.8–90.1]	210
	46–55	544	6	110.3	[40.6–238.5]	91
	56–64	158	2	126.6	[15.4–449.8]	79

Male	All ages	36,423	48	13.2	[9.7–17.5]	759
	0–5	14,200	2	1.4	[0.2–5.1]	7,100
	6–15	18,979	22	11.6	[7.3–17.5]	863
	(0–20)	(33,619)	(26)	(7.7)	(5.1–11.3)	(1,293)
	16–25	695	4	57.6	[15.7–146.7]	174
	26–35	1,156	7	60.6	[24.4–124.4]	165
	36–45	974	6	61.6	[22.6–133.6]	162
	46–55	343	5	145.8	[47.5–336.9]	69
	56–64	76	2	263.2	[32.0–918.5]	38

Female	All ages	31,689	54	17.0	[12.8–22.2]	587
	0–5	11,799	3	2.5	[0.5–7.4]	3,933
	6–15	16,616	27	16.2	[10.7–23.6]	615
	(0–20)	(28,932)	(31)	(10.7)	(7.3–15.2)	(933)
	16–25	823	6	72.9	[26.8–158.0]	137
	26–35	1,249	14	112.1	[61.4–187.4]	89
	36–45	919	3	32.6	[6.7–95.1]	306
	46–55	201	1	49.8	[1.3–274.1]	201
	56–64	82	0	0.0	[0.0–439.9]	na

na: not analyzable.

The findings for the combined age group 0 to 20 years are shown in parentheses.

## DISCUSSION

In this study, we identified the incidence of mumps deafness among mumps patients in Japan, using health insurance claims data from 2005 to 2017. The incidence of mumps deafness was 15.0 cases per 10,000 mumps patients.

Mumps is classified as a Category V infectious disease; under Japan’s Act on the Prevention of Infectious Diseases and Medical Care for Patients with Infectious Diseases, sentinel surveillance is conducted for diseases in this category.^12^ About 3,000 pediatric sentinel sites in Japan report mumps cases each week.^12^ Additionally, a study funded by a Grant-in-Aid for Research from the Ministry of Health, Labour and Welfare found that from 0.431 million to 1.356 million suspected pediatric mumps cases occur each year in Japan.^13^ On the basis of our findings on the incidence of mumps deafness among pediatric mumps patients, approximately 378 to 1,189 patients per year in Japan are estimated to develop mumps deafness.

Mumps is a vaccine-preventable disease, and basic immunization is among the main countermeasures to combat the disease. However, although mumps vaccination is part of the basic vaccination programs in over 120 countries, it is not part of the regular vaccination program in Japan.^14^ Including mumps vaccination in Japan’s regular vaccination program would result in a decline in mumps—and consequently in mumps deafness cases.

In our study, the incidence of mumps deafness was significantly higher among adolescents and adults with mumps than among pediatric patients. To the best of our knowledge, this study was the first to report an 8.4 times higher incidence of mumps deafness per 10,000 mumps cases among 16–64-year-olds than among 0–15-year-olds. This result should be interpreted with caution. Differences in mumps awareness between pediatric patients and adults, for example, should be considered. Further studies are needed to clarify our results. However, at minimum, our study results demonstrate that patients of all ages—not only pediatric patients—are at risk of mumps deafness.

Our study population comprised 5,493,650 cases (about 4% of the total population of Japan) aged 0–64 years. Our data did not include people aged ≥65 years. The retirement age in Japan is approximately 60–65 years. Following retirement, people usually opt out of employment insurance and apply for government health insurance instead. Therefore, employment-based health insurance claims data do not cover the older population. Additionally, the estimate of average income in the present study may have been biased. Average income is associated with education level, which affects factors such as disease awareness and vaccination rate. This point should be taken into account when considering the extent to which the findings from this study can be generalized. A comparison of the present findings with those from the National Database, which contains government health insurance claims and includes many older adults, would be helpful to understand the effects of any bias. Another limitation is that no vaccination record data were available in the claims database used in this study because vaccination is not covered by health insurance. Thus, although vaccination is an important factor, we could not evaluate the vaccination rate.

The Kinki Ambulatory Pediatrics Study Group (KAPSG) investigated the incidence of mumps deafness at 40 pediatric outpatient clinics from 2004 to 2006. Doctors at all 40 clinics were members of the KAPSG. Experienced pediatricians carried out the clinical diagnoses. They reported that 7 of 7,400 patients with mumps aged ≤20 years developed mumps deafness (1 in 1,057 patients with mumps).^4^ In our data, 57 cases of mumps deafness were identified among 62,551 mumps cases for individuals aged 0–20 years (Table 1). This corresponds to an incidence of 1 in 1,097 mumps cases, which is similar to the findings in the KAPSG study; the findings from the two studies did not differ significantly (*P* = 0.598). We therefore believe that our diagnostic definition of mumps deafness was reliable. In conclusion, we believe that the estimates of mumps deafness presented here are accurate.

In general, analyses using insurance claims data have several limitations because the diagnosis criteria are not standardized across providers and institutions. The possibility of underestimation in the present study can be summarized with the following three points. First, some cases lacked clinical test information in the health insurance reimbursement data. Some medical institutes made reimbursements requests using total payments for outpatient pediatric medical services, including clinical tests. In these cases, patients diagnosed with mumps deafness would not meet the disease criteria for mumps deafness in the present study. Second, there is usually low self-awareness of unilateral deafness in pediatric patients (especially those aged <5 years), and it is also difficult for their guardians to be aware of this condition. Our results suggest a relationship between age and mumps deafness, but, considering the possibility of underestimation among pediatric patients, this finding should be interpreted with caution. Another important factor is the effect of inapparent mumps virus infection, which accounts for 30% to 35% of all cases of mumps virus infection.^2^ Analyzing serum specimens from 53 patients with sudden hearing loss, Nomura et al reported that three patients (5%) were positive for mumps immunoglobulin M antibodies.^15^ Okamoto et al also analyzed serum specimens from 130 patients with sudden hearing loss and found that nine patients (6.9%) were positive for mumps immunoglobulin M antibodies.^16^ These figures suggest that 5% to 6.9% of patients with sudden hearing loss have mumps deafness; therefore, some cases of mumps deafness may have been overlooked in the present study.

However, the possibility of overestimation should also be considered. The differential diagnosis of congenital deafness and unilateral mumps deafness in pediatric patients is not easy. Takagi reported that the prevalence of congenital unilateral deafness (serious unilateral deafness) found by neonatal auditory screening was 0.067% (97 cases identified among 145,672 neonates).^17^ Our study subjects were from a wide range of generations, including the generation born before the implementation of this kind of neonatal screening. We therefore cannot rule out the possibility that some cases of congenital unilateral deafness that happened to be identified after mumps infection were included.

In the present study, we reported the incidence of mumps deafness among mumps patients in Japan. Our findings suggested that adult patients, in addition to pediatric patients, are at risk of mumps deafness. Future studies should clarify the relationship between mumps deafness and age.

## APPENDIX A. SUPPLEMENTARY DATA

eTable 1. Definition of clinical condition

eTable 2. Definition of laboratory tests

eFigure 1. Definition of mumps deafness
