# Supplementary material for: Incidence of Mumps Deafness in Japan, 2005–2017: Analysis of Japanese Insurance Claims Database
Source: J Epidemiol. 2022 Jan 5;32(1):21–6. doi: 10.2188/jea.JE20200233 (PMC8666313; doi:10.2188/jea.JE20200233)
Supplement: Supplementary file 1 [file je-32-021-s001.pdf]

eTable 1. Definition of clinical condition

Clinical condition	ICD-10[a]				ICD-10-based Standard Disease Code[b]		
	ICD-10 second classification code	Second classification of disease	ICD-10 third classification code	Third classification of disease	Classified ID	Exchange code	Standard disease name
Mumps deafness	B26	Mumps	B26.8	Mumps with other complications	20053371	SP42	Mumps deafness
Acute sensorineural deafness	H90	Conductive and sensorineural hearing loss	H90.3	Sensorineural hearing loss, bilateral	20098025	FTC3	Bilateral high-tone sloping sensorineural hearing loss
	H90	Conductive and sensorineural hearing loss	H90.3	Sensorineural hearing loss, bilateral	20098024	LNBH	Bilateral high-tone sudden drop sensorineural hearing loss
	H90	Conductive and sensorineural hearing loss	H90.3	Sensorineural hearing loss, bilateral	20077692	MMB7	Bilateral sensorineural hearing loss
	H90	Conductive and sensorineural hearing loss	H90.4	Sensorineural hearing loss, unilateral with unrestricted hearing on the contralateral side	20054502	TH3N	Unilateral sensorineural hearing loss
	H90	Conductive and sensorineural hearing loss	H90.5	Sensorineural hearing loss, unspecified	20079074	N10A	Cochlear nerve deafness
	H90	Conductive and sensorineural hearing loss	H90.5	Sensorineural hearing loss, unspecified	20076891	M9NQ	Labyrinthine deafness
	H90	Conductive and sensorineural hearing loss	H90.5	Sensorineural hearing loss, unspecified	20066048	QMH2	Nervous deafness
	H90	Conductive and sensorineural hearing loss	H90.5	Sensorineural hearing loss, unspecified	20066139	K8J6	Progressive deafness
	H90	Conductive and sensorineural hearing loss	H90.5	Sensorineural hearing loss, unspecified	20056854	CQAQ	Sensorineural hearing loss
	H91	Other hearing loss	H91.2	Sudden idiopathic hearing loss	20072135	FLRN	Sudden deafness
	H91	Other hearing loss	H91.9	Hearing loss, unspecified	20061652	JK5H	Profound deafness
	H91	Other hearing loss	H91.9	Hearing loss, unspecified	20075882	AQHP	Unilateral deafness
Congenital deafness	F80	Specific developmental disorders of speech and language	F80.2	Receptive language disorder	20067696	E5J1	Congenital auditory imperception
	H90	Conductive and sensorineural hearing loss	H90.5	Sensorineural hearing loss, unspecified	20067729	GPV5	Congenital deafness
	H90	Conductive and sensorineural hearing loss	H90.5	Sensorineural hearing loss, unspecified	20067843	G3LF	Congenital deafness
Delayed endolymphatic hydrops	H81	Disorders of vestibular function	H81.0	Meniere's disease	20101275	VM8P	Delayed endolymphatic hydrops
Cochlear Meniere's syndrome	H81	Disorders of vestibular function	H81.0	Meniere's disease	20098854	UA58	Cochlear Meniere's syndrome
Meniere's syndrome	H81	Disorders of vestibular function	H81.0	Meniere's disease	20053401	NCVR	Meniere's syndrome
Meniere's disease	H81	Disorders of vestibular function	H81.0	Meniere's disease	20087640	TUAC	Meniere's disease
	H81	Disorders of vestibular function	H81.0	Meniere's disease	20072146	J0N6	Endolymphatic hydrops
	H81	Disorders of vestibular function	H81.0	Meniere's disease	20072185	Q70R	Inner ear vertigo
	H81	Disorders of vestibular function	H81.0	Meniere's disease	20068157	GS5Q	Vestibular Meniere's disease

Stenosis of Eustachian tube	H68	Eustachian salpingitis and obstruction	H68.1	Obstruction of Eustachian tube	20063320	BCEF	Compression of Eustachian tube
	H68	Eustachian salpingitis and obstruction	H68.1	Obstruction of Eustachian tube	20063325	LU0N	Stenosis of Eustachian tube
	H68	Eustachian salpingitis and obstruction	H68.1	Obstruction of Eustachian tube	20063331	NPM5	Eustachian tube obstruction
Ear herpes	B00	Herpesviral [herpes simplex] infections	B00.1	Herpesviral vesicular dermatitis	20053049	SU33	Herpesviral otitis externa
	B00	Herpesviral [herpes simplex] infections	B00.1	Herpesviral vesicular dermatitis	20063249	E0LA	Ear herpes
Otitis media with effusion	H65	Nonsuppurative otitis media	H65.0	Acute serous otitis media	20058665	FBTU	Acute exudative otitis media
	H65	Nonsuppurative otitis media	H65.2	Chronic serous otitis media	20076669	MLSM	Chronic exudative otitis media
	H65	Nonsuppurative otitis media	H65.9	Nonsuppurative otitis media, unspecified	20078364	LQPR	Otitis media with effusion
	A18	Tuberculosis of other organs	A18.6	Tuberculosis of ear	20059892	VFDM	Tuberculous otitis media
	A38	Scarlet fever	A38	Scarlet fever	20078388	U7C0	Scarlatina otitis media
	B05	Measles	B05.3	Measles complicated by otitis media	20076185	NUA9	Measles otitis media
	H65	Nonsuppurative otitis media	H65.1	Other acute nonsuppurative otitis media	20053814	SDTE	Subacute allergic otitis media
	H65	Nonsuppurative otitis media	H65.1	Other acute nonsuppurative otitis media	20053815	EE8H	Subacute mucoid otitis media
	H65	Nonsuppurative otitis media	H65.1	Other acute nonsuppurative otitis media	20053881	UQKR	Subacute serous mucinous otitis media
	H65	Nonsuppurative otitis media	H65.1	Other acute nonsuppurative otitis media	20053839	TNQE	Subacute bloody otitis media
	H65	Nonsuppurative otitis media	H65.1	Other acute nonsuppurative otitis media	20058158	PSJT	Acute allergic otitis media
	H65	Nonsuppurative otitis media	H65.1	Other acute nonsuppurative otitis media	20058180	FGMS	Acute mucoid otitis media
	H65	Nonsuppurative otitis media	H65.1	Other acute nonsuppurative otitis media	20058662	S20F	Acute serous mucinous otitis media
	H65	Nonsuppurative otitis media	H65.1	Other acute nonsuppurative otitis media	20058335	RBFR	Acute sanguineous otitis media
	H65	Nonsuppurative otitis media	H65.1	Other acute nonsuppurative otitis media	20058600	DJ23	Acute non-suppurative otitis media
	H65	Nonsuppurative otitis media	H65.2	Chronic serous otitis media	20076668	ALKN	Chronic serous otitis media
	H65	Nonsuppurative otitis media	H65.2	Chronic serous otitis media	20076477	NLC9	Chronic tubotympanic catarrh
	H65	Nonsuppurative otitis media	H65.3	Chronic mucoid otitis media	20051754	CTG5	Glue ear
	H65	Nonsuppurative otitis media	H65.3	Chronic mucoid otitis media	20076315	GPMV	Chronic mucoid otitis media
	H65	Nonsuppurative otitis media	H65.4	Other chronic nonsuppurative otitis media	20093167	HNJD	Eosinophilic otitis media

Otitis media	H65	Nonsuppurative otitis media	H65.4	Other chronic nonsuppurative otitis media	20076303	JJ0F	Chronic allergic otitis media
	H65	Nonsuppurative otitis media	H65.4	Other chronic nonsuppurative otitis media	20076667	A933	Chronic serous mucinous otitis media
	H65	Nonsuppurative otitis media	H65.4	Other chronic nonsuppurative otitis media	20076607	U0LK	Chronic non-suppurative otitis media
	H65	Nonsuppurative otitis media	H65.9	Nonsuppurative otitis media, unspecified	20051084	D2LC	Allergic otitis media
	H65	Nonsuppurative otitis media	H65.9	Nonsuppurative otitis media, unspecified	20053348	S733	Mucoid otitis media
	H65	Nonsuppurative otitis media	H65.9	Nonsuppurative otitis media, unspecified	20053345	GLJ2	Mucosus otitis
	H65	Nonsuppurative otitis media	H65.9	Nonsuppurative otitis media, unspecified	20070482	UA1T	Late effects of otitis media
	H65	Nonsuppurative otitis media	H65.9	Nonsuppurative otitis media, unspecified	20074383	EP5N	Non-suppurative otitis media
	H65	Nonsuppurative otitis media	H65.9	Nonsuppurative otitis media, unspecified	20063329	RDG9	Tubotympanitis
	H66	Suppurative and unspecified otitis media	H66.0	Acute suppurative otitis media	20058256	V9VJ	Acute suppurative otitis media
	H66	Suppurative and unspecified otitis media	H66.0	Acute suppurative otitis media	20066422	AKGH	Bullous otitis media
	H66	Suppurative and unspecified otitis media	H66.0	Acute suppurative otitis media	20051729	GBDL	Gradenigo's syndrome
	H66	Suppurative and unspecified otitis media	H66.1	Chronic tubotympanic suppurative otitis media	20076478	SVPB	Chronic tubotympanic suppurative otitis media
	H66	Suppurative and unspecified otitis media	H66.1	Chronic tubotympanic suppurative otitis media	20077769	J5BD	Benign chronic suppurative otitis media
	H66	Suppurative and unspecified otitis media	H66.2	Chronic atticoantral suppurative otitis media	20076489	BR5R	Chronic atticoantral suppurative otitis media
	H66	Suppurative and unspecified otitis media	H66.2	Chronic atticoantral suppurative otitis media	20064845	TVR0	Attic suppuration
	H66	Suppurative and unspecified otitis media	H66.3	Other chronic suppurative otitis media	20076361	NN46	Chronic suppurative otitis media
	H66	Suppurative and unspecified otitis media	H66.3	Other chronic suppurative otitis media	20076360	SHQJ	Chronic suppurative perforative otitis media
	H66	Suppurative and unspecified otitis media	H66.4	Suppurative otitis media, unspecified	20055498	K24L	Suppurative otitis media
	H66	Suppurative and unspecified otitis media	H66.9	Otitis media, unspecified	20070481	ESB5	Otitis media
	H66	Suppurative and unspecified otitis media	H66.9	Otitis media, unspecified	20070483	M4QQ	Facial palsy due to otitis media
	H66	Suppurative and unspecified otitis media	H66.9	Otitis media, unspecified	20062250	QECF	Recurrent otitis media
	H66	Suppurative and unspecified otitis media	H66.9	Otitis media, unspecified	20064190	A68F	Hemorrhagic otitis media
	H66	Suppurative and unspecified otitis media	H66.9	Otitis media, unspecified	20070080	STN3	Simple otitis media
	H66	Suppurative and unspecified otitis media	H66.9	Otitis media, unspecified	20056324	L3E8	Traumatic otitis media

	H66	Suppurative and unspecified otitis media	H66.9	Otitis media, unspecified	20056319	B25T	Traumatic perforative otitis media
	H66	Suppurative and unspecified otitis media	H66.9	Otitis media, unspecified	20058524	SUN4	Acute otitis media
	H66	Suppurative and unspecified otitis media	H66.9	Otitis media, unspecified	20076545	QJ82	Chronic otitis media
	H66	Suppurative and unspecified otitis media	H66.9	Otitis media, unspecified	20076547	UB9N	Late effects of chronic otitis media
	H66	Suppurative and unspecified otitis media	H66.9	Otitis media, unspecified	20076546	SRHN	Chronic otitis media acute exacerbation
	H66	Suppurative and unspecified otitis media	H66.9	Otitis media, unspecified	20076548	AQCR	Exacerbation after surgery for chronic otitis media
	H66	Suppurative and unspecified otitis media	H66.9	Otitis media, unspecified	20076519	HMRK	Chronic perforative otitis media
	H66	Suppurative and unspecified otitis media	H66.9	Otitis media, unspecified	20065773	H3HQ	Neonatal otitis media
	H66	Suppurative and unspecified otitis media	H66.9	Otitis media, unspecified	20067965	SSBK	Perforative otitis media
	H66	Suppurative and unspecified otitis media	H66.9	Otitis media, unspecified	20064263	U9GV	Postoperative otitis media
	H66	Suppurative and unspecified otitis media	H66.9	Otitis media, unspecified	20064268	RUAJ	Postoperative chronic otitis media
	H66	Suppurative and unspecified otitis media	H66.9	Otitis media, unspecified	20071054	C2M1	Old otitis media
	H66	Suppurative and unspecified otitis media	H66.9	Otitis media, unspecified	20060674	SAFH	Hydrotympanum
	H71	Cholesteatoma of middle ear	H71	Cholesteatoma of middle ear	20051873	RPVA	Cholesterin otitis media
	H71	Cholesteatoma of middle ear	H71	Cholesteatoma of middle ear	20065913	CQND	Chronic suppurative otitis media with cholesteatoma
	H74	Other disorders of middle ear and mastoid	H74.1	Adhesive middle ear disease	20077318	GLEJ	Adhesive otitis media
	H83	Other diseases of inner ear	H83.0	Labyrinthitis	20070484	HT38	Labyrinthitis due to otitis media
	J11	Influenza, virus not identified	J11.8	Influenza with other manifestations, virus not identified	20051181	UFVR	Influenzal otitis media
	T70	Effects of air pressure and water pressure	T70.0	Otitic barotrauma	20057858	EQQ1	Barotitis media
	T70	Effects of air pressure and water pressure	T70.0	Otitic barotrauma	20061468	H69V	Aerotitis media
Congenital middle ear malformation	Q16	Congenital malformations of ear causing impairment of hearing	Q16.4	Other congenital malformations of middle ear	20070494	QK0C	Congenital middle ear malformation
	C07	Malignant neoplasm of parotid gland	C07	Malignant neoplasm of parotid gland	20063262	N3D5	Parotid cancer
	C30	Malignant neoplasm of nasal cavity and middle ear	C30.1	Malignant neoplasm of nasal cavity and middle ear	20063323	BOF9	Auditory tube cancer
	C30	Malignant neoplasm of nasal cavity and middle ear	C30.1	Malignant neoplasm of nasal cavity and middle ear	20070480	R2H2	Malignant middle ear tumor
	C30	Malignant neoplasm of nasal cavity and middle ear	C30.1	Malignant neoplasm of nasal cavity and middle ear	20102064	TNTL	Endolymphatic sac tumor

Acoustic neuroma	C30	Malignant neoplasm of nasal cavity and middle ear	C30.1	Malignant neoplasm of nasal cavity and middle ear	20072179	TNN1	Inner ear cancer
	C49	Malignant neoplasm of other connective and soft tissue	C49.0	Connective and soft tissue of head, face and neck	20095158	NSJQ	Mandibular rhabdomyosarcoma
	C49	Malignant neoplasm of other connective and soft tissue	C49.0	Connective and soft tissue of head, face and neck	20063275	E2CA	Subaural sarcoma
	C49	Malignant neoplasm of other connective and soft tissue	C49.0	Connective and soft tissue of head, face and neck	20095132	K7CF	Neck malignant fibrous histiocytoma
	C49	Malignant neoplasm of other connective and soft tissue	C49.0	Connective and soft tissue of head, face and neck	20093691	LQF5	Malignant neck soft tissue tumor
	C49	Malignant neoplasm of other connective and soft tissue	C49.0	Connective and soft tissue of head, face and neck	20095160	MS82	Cervical rhabdomyosarcoma
	C49	Malignant neoplasm of other connective and soft tissue	C49.0	Connective and soft tissue of head, face and neck	20095188	J20Q	Cervical synovial sarcoma
	C49	Malignant neoplasm of other connective and soft tissue	C49.0	Connective and soft tissue of head, face and neck	20059752	FR1V	Cervical sarcoma
	C49	Malignant neoplasm of other connective and soft tissue	C49.0	Connective and soft tissue of head, face and neck	20092734	R8VM	Cervical liposarcoma
	C49	Malignant neoplasm of other connective and soft tissue	C49.0	Connective and soft tissue of head, face and neck	20095182	RQPQ	Cervical angiosarcoma
	C49	Malignant neoplasm of other connective and soft tissue	C49.0	Connective and soft tissue of head, face and neck	20095163	MJH6	Buccal rhabdomyosarcoma
	C49	Malignant neoplasm of other connective and soft tissue	C49.0	Connective and soft tissue of head, face and neck	20095181	MCBA	Buccal angiosarcoma
	C49	Malignant neoplasm of other connective and soft tissue	C49.0	Connective and soft tissue of head, face and neck	20095134	VC12	Head malignant fibrous histiocytoma
	C49	Malignant neoplasm of other connective and soft tissue	C49.0	Connective and soft tissue of head, face and neck	20095162	DV75	Head rhabdomyosarcoma
	C49	Malignant neoplasm of other connective and soft tissue	C49.0	Connective and soft tissue of head, face and neck	20095190	R08S	Head synovial sarcoma
	C49	Malignant neoplasm of other connective and soft tissue	C49.0	Connective and soft tissue of head, face and neck	20092863	CLCR	Head liposarcoma
	C49	Malignant neoplasm of other connective and soft tissue	C49.0	Connective and soft tissue of head, face and neck	20095184	EN2H	Head angiosarcoma
	C49	Malignant neoplasm of other connective and soft tissue	C49.0	Connective and soft tissue of head, face and neck	20071862	CSSJ	Malignant head soft tissue tumor
	C49	Malignant neoplasm of other connective and soft tissue	C49.0	Connective and soft tissue of head, face and neck	20095159	M76T	Facial rhabdomyosarcoma
	C72	Malignant neoplasm of spinal cord, cranial nerves, and other parts of central nervous system	C72.4	Malignant neoplasm , auditory nerve of the part of spinal cord, cranial nerve and other central nervous system	20079584	T0E3	Auditory nerve glioma

[a] *International Statistical Classification of Diseases and Related Health Problems 10th Revision* (ICD-10)-WHO Version for 2016

[b] http://byomei.org/Scripts/ICD10Categories/default2_ICD.asp?CategoryID=1 ; Accessed 24.06.2020.

eTable 2. Definition of laboratory tests

Laboratory test	Health care fee code	Code*	Code description	Revision year
Viral antibody titer according to immunoglobulin class (mumps virus)	D012	160157310	Viral antibody titer according to immunoglobulin class (mumps virus), detailed	2004
	D012	160157310	Viral antibody titer according to immunoglobulin class (mumps virus), detailed	2006
	D012	160157310	Viral antibody titer according to immunoglobulin class (mumps virus), detailed	2008
	D012	160157310	Viral antibody titer according to immunoglobulin class (mumps virus)	2010
	D012	160157310	Viral antibody titer according to immunoglobulin class (mumps virus)	2012
	D012	160157310	Viral antibody titer according to immunoglobulin class (mumps virus)	2014
	D012	160157310	Viral antibody titer according to immunoglobulin class (mumps virus)	2016
Viral antibody titer (mumps virus)	D012	160042410	Viral antibody titer (mumps virus)	2004
	D012	160042410	Viral antibody titer (mumps virus)	2006
	D012	160042410	Viral antibody titer (mumps virus)	2008
	D012	160042410	Viral antibody titer, semi-quantitative (mumps virus)	2010
	D012	160042410	Viral antibody titer, qualitative/semi-quantitative/quantitative (mumps virus)	2012
	D012	160042410	Viral antibody titer, qualitative/semi-quantitative/quantitative (mumps virus)	2014
	D012	160042410	Viral antibody titer, qualitative/semi-quantitative/quantitative (mumps virus)	2016
Subjective audiometry, pure tone audiometry	D244	160078010	Subjective audiometry, pure tone audiometry	2004
	D244	160078010	Subjective audiometry, pure tone audiometry	2006
	D244	160078010	Subjective audiometry, pure tone audiometry	2008
	D244	160078010	Subjective audiometry, pure tone audiometry	2010
	D244	160078010	Subjective audiometry, pure tone audiometry	2012
	D244	160078010	Subjective audiometry, pure tone audiometry	2014
	D244	160078010	Subjective audiometry, pure tone audiometry	2016

*Codes of medical service fees (tests) listed by the Ministry of Health, Labour and Welfare

eFigure 1. Definition of mumps deafness

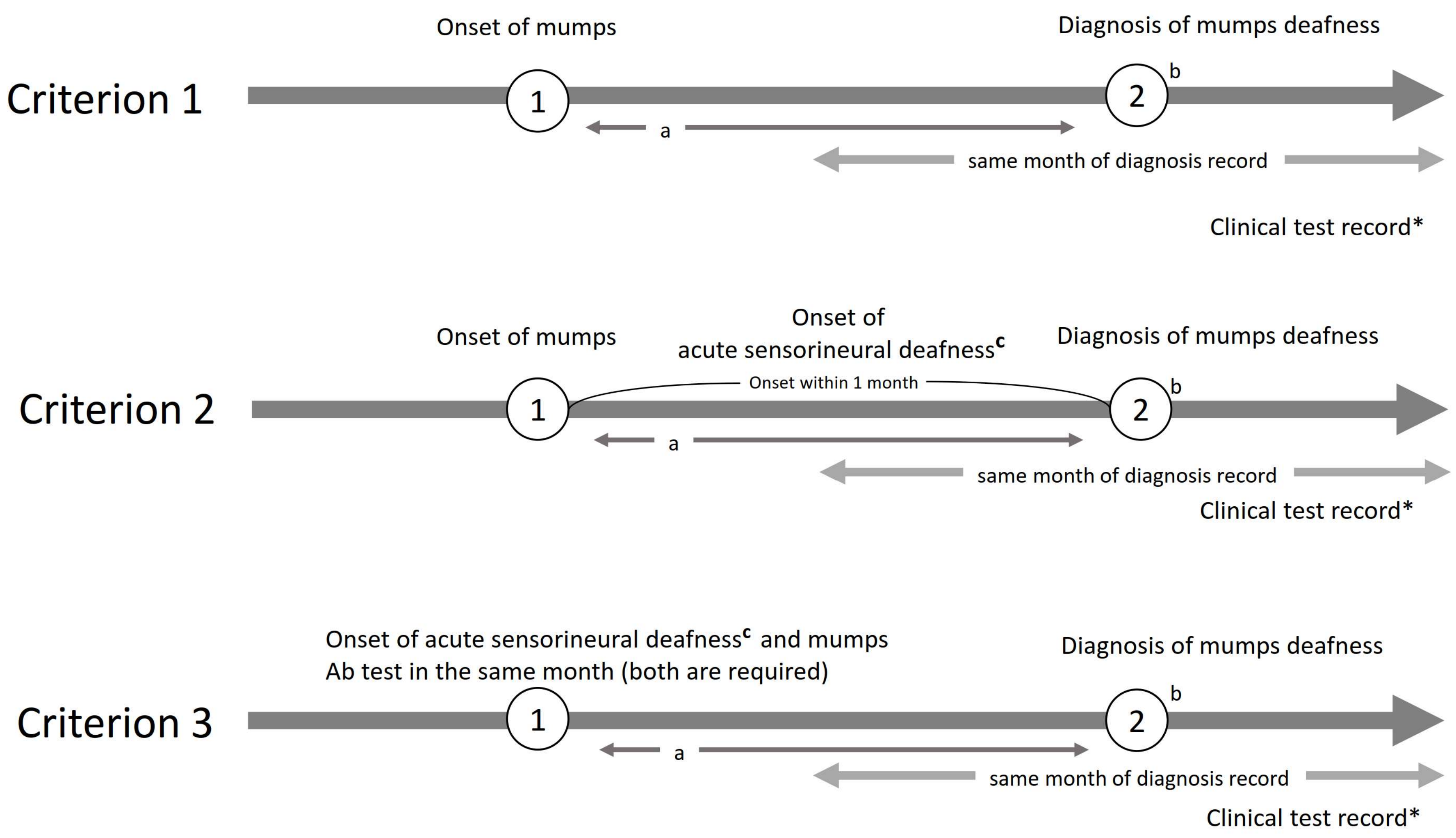

eFigure 1. Definition of mumps deafness

In this study, after excluding cases that matched our exclusion criteria (a, b, and c), three criteria were used to define mumps deafness. Mumps deafness was defined as cases meeting one of these three criteria. We added Criteria 2 and 3 because mumps deafness may be misdiagnosed as acute sensorineural deafness.

Criterion 1: Mumps deafness diagnosis was used to define mumps deafness.

Criterion 2: Acute sensorineural deafness within 1 month after mumps onset was used to define mumps deafness.

Criterion 3: Onset of acute sensorineural deafness and mumps Ab test to confirm mumps infection were used to define mumps deafness.

*The diagnosis of mumps deafness was made by viral antibody titer determination according to immunoglobulin class (mumps virus) and/or standard pure tone audiometry.

[a]Excluding patients with a documented diagnosis of congenital deafness, a documented diagnosis of mumps deafness, or acute sensorineural deafness within 3 months after the start of observation

[b]Excluding patients with a documented diagnosis of Meniere's syndrome, Meniere's disease, ear herpes, Eustachian tube obstruction, delayed endolymphatic hydrops, otitis media, acoustic neuroma, congenital inner ear malformation, exudative otitis media, or cochlear Meniere's disease in the same month

[c]Acute sensorineural deafness was defined as a documented diagnosis of bilateral sensorineural hearing loss, bilateral high-tone sudden drop sensorineural hearing loss, bilateral high-tone sloping sensorineural hearing loss, unilateral sensorineural hearing loss, sensorineural hearing loss, nervous deafness, cochlear nerve deafness, labyrinthine deafness, progressive deafness, unilateral deafness, sudden deafness, or profound deafness.
